# An Approach for Identifying Cytokines Based on a Novel Ensemble Classifier

**DOI:** 10.1155/2013/686090

**Published:** 2013-08-21

**Authors:** Quan Zou, Zhen Wang, Xinjun Guan, Bin Liu, Yunfeng Wu, Ziyu Lin

**Affiliations:** ^1^School of Information Science and Technology, Xiamen University, Xiamen, Fujian, China; ^2^Center for Cloud Computing and Big Data, Xiamen University, Xiamen, Fujian, China; ^3^Shanghai Key Laboratory of Intelligent Information Processing, Shanghai, China; ^4^School of Computer Science and Technology, Harbin Institute of Technology Shenzhen Graduate School, Shenzhen, Guangdong, China

## Abstract

Biology is meaningful and important to identify cytokines and investigate their various functions and biochemical mechanisms. However, several issues remain, including the large scale of benchmark datasets, serious imbalance of data, and discovery of new gene families. In this paper, we employ the machine learning approach based on a novel ensemble classifier to predict cytokines. We directly selected amino acids sequences as research objects. First, we pretreated the benchmark data accurately. Next, we analyzed the physicochemical properties and distribution of whole amino acids and then extracted a group of 120-dimensional (120D) valid features to represent sequences. Third, in the view of the serious imbalance in benchmark datasets, we utilized a sampling approach based on the synthetic minority oversampling technique algorithm and K-means clustering undersampling algorithm to rebuild the training set. Finally, we built a library for dynamic selection and circulating combination based on clustering (LibD3C) and employed the new training set to realize cytokine classification. Experiments showed that the geometric mean of sensitivity and specificity obtained through our approach is as high as 93.3%, which proves that our approach is effective for identifying cytokines.

## 1. Introduction

Cytokines are proteins or micromolecular polypeptides mainly secreted by immune cells. They play an important regulatory role in many cellular activities, such as growth, differentiation, and interactions between cells. Research on cytokine identification and classification has important theoretical and practical significance that may assist in the elucidation of immune regulatory mechanisms at the molecular level and contribute to disease prevention, diagnosis, and treatment. The classification and identification of proteins are of great importance in the postgenomic era. Since the 1990s, with the evolution of the human genome project, studies on biological information excavation have developed rapidly, and large numbers of protein sequences have been obtained. The scale of original bioinformatics data has grown rapidly and continues to double every ten months [1]. At present, protein classification is based mostly on their structures and functions in molecular biology [2]; thus, more information on protein classification and prediction is necessary. Cytokines are a type of proteins produced by immunocytes or related cells that regulate the functions of certain cells. They play important roles in many physiological activities. Only through accurate classification and recognition to the original sequences of cytokines can the structure and functions of unknown types of cytokines be understood. Such information will contribute to future endeavors to detect the nature of diseases at the molecular level and prevent, diagnose, and treat human diseases.

The major biological laboratories in the world have predicted the classification of all kinds of genes, protein structures, and their functions by artificial experiments. The basic method used to identify cytokines involves obtaining their sequence structures and functions by manual prediction [1], which can yield small-scale data. However, this approach is inappropriate when the data is large. Several methods for cytokines identification have emerged over the last two decades. These methods include (1) hidden Markov model (HMM) [3, 4] and artificial neutral network (ANN) [5–7], which is based on statistical learning theory but presents significant limitations for finite sample processing; (2) Basic Local Alignment Search Tool (BLAST) [8] and FASTA [9, 10], which are approaches that utilize sequence alignments based on similarity but can only effectively identify and classify the sequences of homologous structures; (3) CTKPred, a method proposed by Huang in 2005 [11] based on support vector machine (SVM); this method extracts the dipeptide composition properties of cytokines and shows improved prediction accuracy; and (4) CytoPred, a method proposed by Lata [12] at the beginning of 2008 based on the PSI-BLAST; while this method yields favorable results, it is also unstable because it relies heavily on samples, and different samples may yield different performance. 

In our approach, we selected amino acids composed of cytokines as research objects. We obtained benchmark datasets from the PFAM [13] database and deleted similar and redundant sequences. We then extracted a group of valid 120-dimensional (120D) features to represent the protein sequences of cytokines. These 120D features are the distribution features of amino acid (AA) with certain physicochemical properties [14], including hydrophobicity, normalized Van der Waals volume, polarity, polarizability, change, surface tension, secondary structure (SS), and solvent accessibility. Because the sequence numbers of positive (cytokines) and negative instances are extremely imbalanced (the number of negative instances is 84 times the number of positive instances), we utilized a sampling approach based on *K*-means clustering the undersampling algorithm [15] and the synthetic minority oversampling technique (SMOTE) oversampling algorithm [16]. We built a library for dynamic selection and circulating combination based on clustering (LibD3C) on the rebuilt training sets to realize cytokine classification. We achieved a success rate of 93.3%, which is higher than the result obtained using Cai's approach [17]. Cai et al. utilized 188D features of the AA composition, such as content, distribution, and bivalent frequency. The experiments prove that our approach effectively achieves cytokine identification. 

Our work shows improved prediction accuracy for large-scale data and extends the prediction range of cytokine families. Compared with prior studies, we not only focused on features extraction but also extended our work to four aspects: accurate pretreatment of the benchmark data, extraction of multidimensional feature vectors [18], rebuilding training sets through the oversampling and undersampling approaches, and adoption of a novel ensemble classifier. 

## 2. Methods

We developed several procedures to achieve cytokine identification and classification.

### 2.1. Data

Cytokine identification refers to the process of determining whether a protein is a cytokine or not. This classification process divides proteins into two categories, cytokines and non cytokines, which are positive and negative instances, respectively.

Due to the low number of cytokines currently available, building a representative and nonredundant negative set is very important. We chose the protein family database (PFAM [13]) based on structural information as the data source and built a negative dataset according to two principles: (1) every negative instance comes from different protein families and is the longest one in its family, and (2) negative instances from positive families cannot be selected.

We downloaded 16245 cytokines from the UniProt (Universal Protein, release 2012_09) [19–21] database website (http://www.uniprot.org/uniprot/) and obtained the family numbers of these cytokines. We removed duplicate numbers and extracted the longest cytokine sequences of their families corresponding to the non-duplicate numbers from PFAM. We obtained 126 representative cytokines as the positive set.

We then excluded positive protein families (126) from the PFAM database (10714) and obtained 10588 negative protein families. We extracted the longest sequences from the negative protein families and obtained 10588 negative instances as the negative set. Positive and negative instances constitute the original imbalanced dataset.

### 2.2. Features Extraction

The developmental direction of protein classification is the extraction of the characteristic properties of protein sequences and determination of the relationships between positions and structural functions in original sequence mode using appropriate mathematical tools. We extracted a group of 120 valid features to represent the protein sequence based on the distribution of AAs with certain physicochemical properties [22]. We adopted *S* = *R*_1_ *R*_2_ *R*_3_ … *R*_*L*_ to represent a protein sequence, where *R*_*i*_ represents the amino acid in position *i* and *L* represents the sequence length, in other words, the number of amino acids. Twenty amino acids are expressed as

(1) $$\begin{array}{c} AA=\left\{A,C,D,E,F,G,H,I,K,L,M,N,P,Q,R,S,T,V,W,Y\right\}. \end{array}$$

#### 2.2.1. Algorithm Based on AA Composition

The algorithm based on AA composition [23] has been previously formulated. By calculating the frequencies of 20 amino acids in the protein sequence and using these frequencies to represent a specific protein sequence, each sequence becomes a 20D vector after features conversion:

(2) $$\begin{array}{c} {\left(v_{1},v_{2},v_{3},\ldots ,v_{20}\right)}^{T}=\left(\frac{n_{1}}{L},\frac{n_{2}}{L},\frac{n_{3}}{L},\ldots ,\frac{n_{20}}{L}\right), \end{array}$$

where *n*_*i*_ (*i* = 1,2, 3,…, 20) represents the quantity of an AA in the protein sequence. Obviously, ∑_*i*=1_^20^ *v*_*i*_ = 1.

#### 2.2.2. Algorithm Based on the Distribution of AAs with Certain Physicochemical Properties

**Figure 1:**
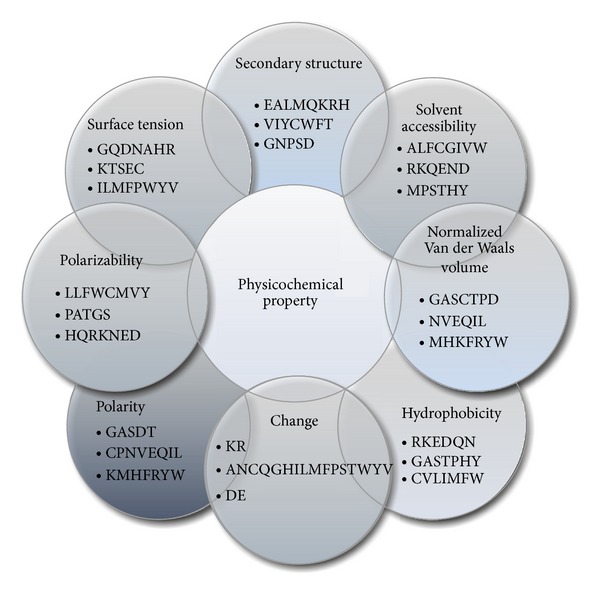
Division of amino acids into 3 different groups by different physicochemical properties.

The nature of AAs is determined by their side chains, and these side chains vary in shape, charge, and hydrophobicity. AAs sequences thus have different structural features and physiological functions. Based on this perspective, we employed eight physicochemical properties [24–29] of AAs such as SS, solvent accessibility, normalized Van der Waals volume, hydrophobicity, change, polarizability, polarity, and surface tension. The eight physicochemical properties and the basis for their division are shown in Figure 1.

We calculated the characteristic value of the distribution of AAs with certain physicochemical properties [29] (*D*). Using SS [26] as an example. 

To the AAs of EALMQKRH group, making the position of the first, 25%, 50%, 75%, and 100% of AAs chain represented by *p*_11_, *p*_12_,…, *p*_15_, respectively, and the lengths from *p*_11_, *p*_12_,…, *p*_15_ to the head of this protein sequence are *DSS*_11_, *DSS*_12_,…, *DSS*_15_, respectively. We can calculate similar parameters of two other AA SS as *DSS*_21_, *DSS*_22_,…, *DSS*_25_, *DSS*_31_, *DSS*_32_,…, *DSS*_35_. *V*_1_, *V*_2_,…, *V*_15_ can then be represented as

(3) $$\begin{array}{c} \left[\begin{array}{ccc} V_{1} & V_{6} & V_{11}\\ V_{2} & V_{7} & V_{12}\\ V_{3} & V_{8} & V_{13}\\ V_{4} & V_{9} & V_{14}\\ V_{5} & V_{10} & V_{15} \end{array}\right]=\left[\begin{array}{ccc} \frac{DSS_{11}}{L} & \frac{DSS_{21}}{L} & \frac{DSS_{31}}{L}\\ \frac{DSS_{12}}{L} & \frac{DSS_{22}}{L} & \frac{DSS_{32}}{L}\\ \frac{DSS_{13}}{L} & \frac{DSS_{23}}{L} & \frac{DSS_{33}}{L}\\ \frac{DSS_{14}}{L} & \frac{DSS_{24}}{L} & \frac{DSS_{34}}{L}\\ \frac{DSS_{15}}{L} & \frac{DSS_{25}}{L} & \frac{DSS_{35}}{L} \end{array}\right]. \end{array}$$

**Figure 2:**
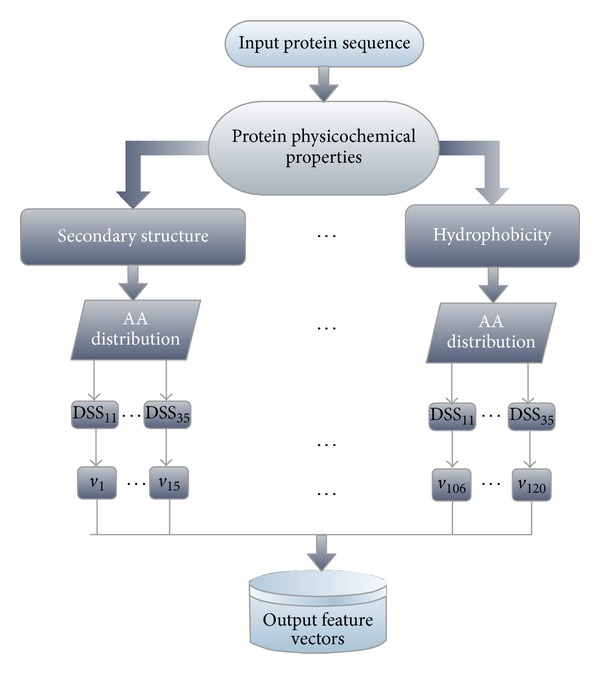
Extraction process of the 120-dimensional (120D) feature vectors (v).

Thus, 15d feature vectors may be extracted from the SS property. We can extract 120D feature vectors after the eight physicochemical properties are analyzed. This process is presented in Figure 2.

In 2003, Cai et al. [17] established a method of features extraction based on the composition and distribution of amino acids combined with their physicochemical properties. A total of 188D features were extracted, including the 120D features we used in this paper (3), 20D features of AA compositions (2), 24d features based on the contents of AAs with certain physicochemical properties (4), and 24d features of bivalent frequency (5) based on the eight physicochemical properties described above. We will demonstrate that the effectiveness of our 120D features is superior to that of the 188D combined features through multiple sets of experiments

(4) $$\begin{array}{c} \left[\begin{array}{ccc} \varphi_{11} & \varphi_{12} & \varphi_{13}\\ \varphi_{21} & \varphi_{22} & \varphi_{23}\\ \varphi_{31} & \varphi_{32} & \varphi_{33}\\ \varphi_{41} & \varphi_{42} & \varphi_{43}\\ \varphi_{51} & \varphi_{52} & \varphi_{53}\\ \varphi_{61} & \varphi_{62} & \varphi_{63}\\ \varphi_{71} & \varphi_{72} & \varphi_{73}\\ \varphi_{81} & \varphi_{82} & \varphi_{83} \end{array}\right]=\left[\begin{array}{ccc} \frac{CSS_{11}}{L} & \frac{CSS_{12}}{L} & \frac{CSS_{13}}{L}\\ \frac{CSS_{21}}{L} & \frac{CSS_{22}}{L} & \frac{CSS_{23}}{L}\\ \frac{CSS_{31}}{L} & \frac{CSS_{32}}{L} & \frac{CSS_{33}}{L}\\ \frac{CSS_{41}}{L} & \frac{CSS_{42}}{L} & \frac{CSS_{43}}{L}\\ \frac{CSS_{51}}{L} & \frac{CSS_{52}}{L} & \frac{CSS_{53}}{L}\\ \frac{CSS_{61}}{L} & \frac{CSS_{62}}{L} & \frac{CSS_{63}}{L}\\ \frac{CSS_{71}}{L} & \frac{CSS_{72}}{L} & \frac{CSS_{73}}{L}\\ \frac{CSS_{81}}{L} & \frac{CSS_{82}}{L} & \frac{CSS_{83}}{L} \end{array}\right], \end{array}$$

(5) $$\begin{array}{c} \left[\begin{array}{ccc} \phi_{11} & \phi_{12} & \phi_{13}\\ \phi_{21} & \phi_{22} & \phi_{23}\\ \phi_{31} & \phi_{32} & \phi_{33}\\ \phi_{41} & \phi_{42} & \phi_{43}\\ \phi_{51} & \phi_{52} & \phi_{53}\\ \phi_{61} & \phi_{62} & \phi_{63}\\ \phi_{71} & \phi_{72} & \phi_{73}\\ \phi_{81} & \phi_{82} & \phi_{83} \end{array}\right]=\left[\begin{array}{ccc} \frac{BSS_{11}}{L} & \frac{BSS_{12}}{L} & \frac{BSS_{13}}{L}\\ \frac{BSS_{21}}{L} & \frac{BSS_{22}}{L} & \frac{BSS_{23}}{L}\\ \frac{BSS_{31}}{L} & \frac{BSS_{32}}{L} & \frac{BSS_{33}}{L}\\ \frac{BSS_{41}}{L} & \frac{BSS_{42}}{L} & \frac{BSS_{43}}{L}\\ \frac{BSS_{51}}{L} & \frac{BSS_{52}}{L} & \frac{BSS_{53}}{L}\\ \frac{BSS_{61}}{L} & \frac{BSS_{62}}{L} & \frac{BSS_{63}}{L}\\ \frac{BSS_{71}}{L} & \frac{BSS_{72}}{L} & \frac{BSS_{73}}{L}\\ \frac{BSS_{81}}{L} & \frac{BSS_{82}}{L} & \frac{BSS_{83}}{L} \end{array}\right]. \end{array}$$

### 2.3. Sampling

Random sampling may miss samples with strong feature prediction capability. To compensate for this shortcoming, we applied the undersampling approach using *K*-means clustering [15]. To avoid extremely sparse numbers of samples in the datasets by undersampling, we generated samples artificially using the SMOTE algorithm [16] to increase the size of the minimum class. The ensemble algorithm of undersampling combined with oversampling not only avoids producing excessive noise but also solves the problem of sample shortage. 

The SMOTE oversampling algorithm and *K*-means undersampling algorithm are illustrated in Algorithms 1 and 2, respectively.

The distances between samples and clustering centroids were measured using the square of the Euclidean distance

(6) $$\begin{array}{c} d=||x_{p}-{\hat{\mu }}_{i}||,\;p=1,2,\ldots ,n;\;i=1,2,\ldots ,\lambda_{n}, \end{array}$$

where *x*_*p*_ represents clustering samples and ${\hat{\mu }}_{i}$ represents clustering centroids.

**Figure 3:**
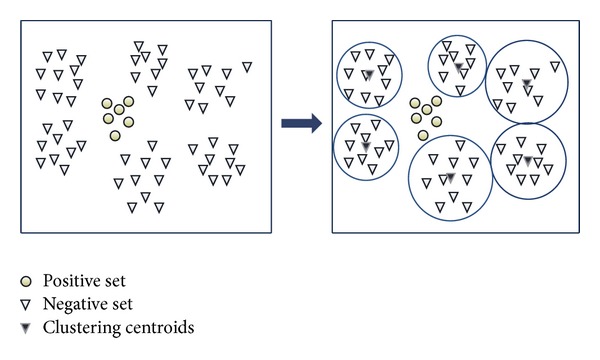
The process of undersampling applies *K*-means clustering.

The process of undersampling by *K*-means clustering is illustrated in Figure 3.

*K*-means clustering is simple and rapid. Its time complexity is *O* (*nkt*), and *n*, *k*, and *t* represent the negative sample size, initial negative cluster size, and iteration, respectively. The initial parameters directly influence the time performance of clustering, and the effective parameters significantly reduce the iterations. 

To solve the problems of missing samples and introducing noise through the ensemble algorithm, we considered oversampling and undersampling to achieve balance. The ensemble algorithm is illustrated in Algorithm 3.

### 2.4. Ensemble Classifier

Ensemble classification is a method used to combine various basic classifiers that each has independent decision-making ability. Generally speaking, the prediction ability of an ensemble classifier is superior to that of a single classifier because the former can address the diversities produced by the latter more efficiently when faced with different problems [30]. According to the principle that the effect of the ensemble classifier is closer to the globally optimal solution than that of the single classifier, we further improved the prediction accuracy of our proposed technique by increasing the diversity of basic classifiers.

**Figure 4:**
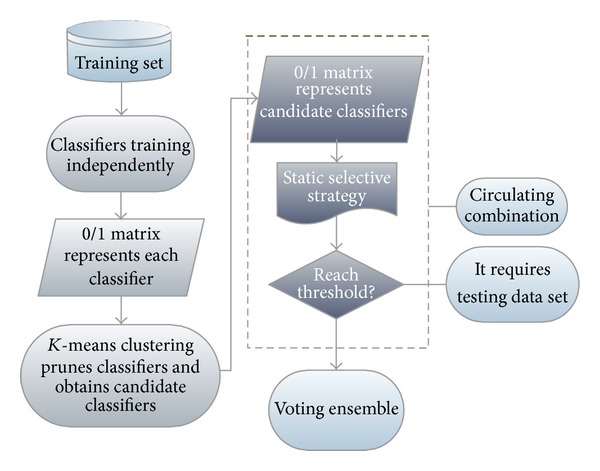
Classifier architecture.

We adopted the *K*-means algorithm [31] to cluster all classification results of basic classifiers, and the diversity of basic classifiers selected from each category was further improved. Classifiers were selected through a circulating combined dynamic selective strategy (Circulatory Ensemble Forward Selection, CEFS), and voted for the last result. The classifier architecture is illustrated in Figure 4.

We utilized 18 basic classifiers to create the training set. The basic classifiers utilized in this study are sequential minimal optimization (SMO), support vector machine (SVM), logistic regression, Instance-based 1 (IB1), Instance-based 5 (IB5), instance-based 10 (IB10), decision table, conjunctive rule, one rule (one *R*), simple cart, JRip, Zero *R*, random tree, naïve Bayes, random forest (RF), decision stump, J48, and functional trees (FT), which are labeled as *C*_1_, *C*_2_,…, *C*_18_, respectively. These basic classifiers were applied to the training set independently, and the training results are represented as

(7) $$\begin{array}{c} R_{ij}=\left\{0,1\right\};\;i=1,2,\ldots ,18;\;j=1,2,\ldots ,m, \end{array}$$

where *m* is the number of training samples. 

If *R*_*ij*_ = 0, the sample *j* is classified wrongly by classifier *i*; otherwise, it is correct. Figure 4 shows the results matrix obtained using the *K*-means clustering algorithm.

We used *K* = 9 as the initial number of clustering centroids in the *K*-means algorithm. These centroids were divided into nine groups based on the training results of basic classifiers. The basic classifiers with the best performance in each cluster were sorted in descending order according to their classification accuracy to form a set of selected classifiers.

**Figure 5:**
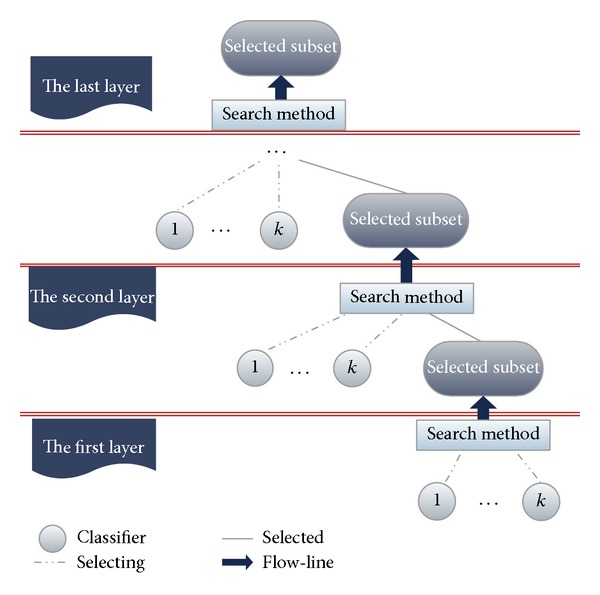
Circulating combination of CEFS.

The classifier combination was processed continuously with the circulating combination methodology to further optimize its effects. We set up a new variable CC (chosen classifier) to store the selected basic classifiers. In each cycle, the CEFS algorithm was employed to basic classifiers continuously to choose the best performing classifiers and create classifiers combination with these classifiers abiding by the vote rule. If the process results in a decline in diversity and an increase in accuracy at the same time, the classifier is added to the CC. This process is considered completed once the accuracy obtained is superior to the initial goal. The detailed algorithm description is illustrated in Figure 5. 

The target accuracy, optimal accuracy, and step were initialized to 1, 0, and 0.05, respectively. The diversity was set to infinity, and the accuracy of classification and number of selected basic classifiers were set to 0.

The ensemble classifier described in this section is highly focused on the selection of basic classifiers. Through comprehensive application of various methods, we integrated the most effective basic classifiers so as to optimize the classification results.

## 3. Experiments

We performed a series of experiments to confirm the effectiveness of our method. First, we analyzed the effectiveness of the extracted 120D feature vectors. Second, we showed the performance of other sampling strategies and compared findings with the performance of the ensemble classifier we developed. Finally, we tested all known proteins and determined 4151 cytokines. These experiments are discussed in detail in this section.

### 3.1. Performance of Evaluation Standards

Sensitivity (SN) (8), specificity (SP) (9), GM (10), and overall accuracy (ACC) (11) are often used to evaluate the results of prediction or classification in bioinformatics

(8) $$\begin{array}{c} \text{SN}=\frac{\text{TP}}{\text{TP}+\text{FN}}, \end{array}$$

(9) $$\begin{array}{c} \text{SP}=\frac{\text{TN}}{\text{TN}+\text{FP}}, \end{array}$$

(10) $$\begin{array}{c} \text{GM}=\sqrt{\text{SN}\times \text{SP}}, \end{array}$$

(11) $$\begin{array}{c} \text{ACC}=\frac{\text{TP}+\text{TN}}{\text{TP}+\text{TN}+\text{FP}+\text{FN}}. \end{array}$$

These four parameters are recognized as reliable measures for evaluating the performance of machine learning methods. TP, TN, FP, and FN represent true positive, true negative, false positive, and false negative, respectively.

Due to the extreme imbalance of positive and negative instances in this paper, the ACC value roughly equaled the SP value (12). Hence, only SN, SP, and GM were adopted as evaluation standards in our study

(12) $$\begin{array}{c} \text{ACC}=\frac{\text{TP}+\text{TN}}{\text{TP}+\text{TN}+\text{FP}+\text{FN}}\approx \frac{\text{TN}}{\text{TN}+\text{FP}}=\text{SP}. \end{array}$$

### 3.2. Performance of Sampling Strategies

The test dataset consisted of 126 positive feature samples and 10588 negative feature samples; thus, it may be considered extremely imbalanced. They are extracted by 120D feature extraction algorithm in agreement with the one mentioned in Section 3.3. After directly performing 10-fold cross-validation on the test dataset without sampling by LibD3C classifier, we achieved an SP value as high as 99.9% but an SN value as low as 0.80% and a GM value of only 8.90%. The effect of that is even worse than random sampling effect. We conducted SMOTE oversampling on the positive set and *K*-means clustering undersampling on the negative set. The rebuilt testing set was balanced and contained 2019 positive feature samples and 1996 negative feature samples. The detailed algorithms refer to Section 2. 

**Figure 6:**
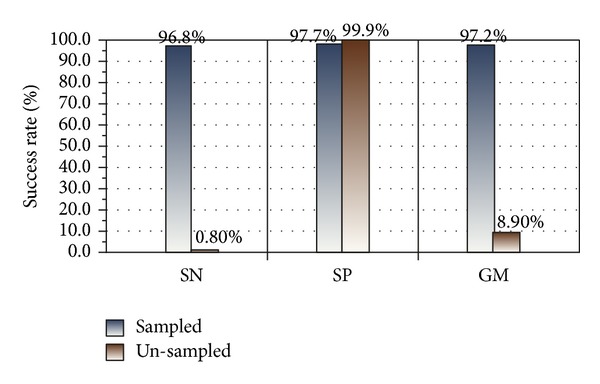
Comparison of validation on sampled dataset and unsampled dataset.

SN, SP, and GM values of classification results obtained from 10-fold cross-validation on the unsampled and sampled datasets are illustrated in Figure 6.

Figure 6 shows that the effect of 10-fold cross-validation on the sampled dataset is quite good. The values of SN, SP, and GM reached 96.8%, 97.7%, and 97.2%, respectively, far better than the training results of the unsampled dataset. These results provide strong evidence that oversampling and undersampling processes on the testing set are necessary.

### 3.3. Performance of 120D Feature Vectors

We extracted 120D feature vectors of positive and negative instances based on the distribution of AAs with certain physicochemical properties. The validity was verified by Experiments 1 and 2.

**Experiment 1**

**Figure 7:**
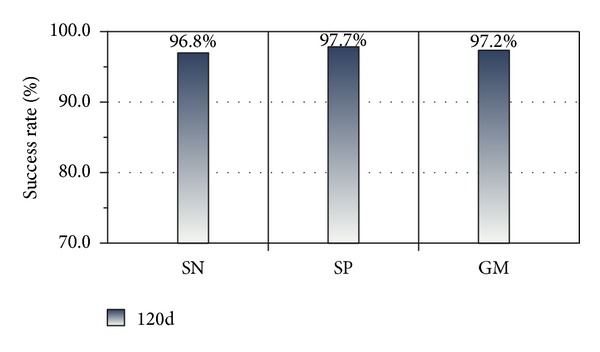
10-fold cross-validation result of training set (120D features).

The sampled dataset with 120D feature was trained, and the results of 10-fold cross-validation were analyzed. The training model was saved as model_1_ by Weka (version 3.7.9). We calculated the SN, SP, and GM values of model_1_ and illustrated the results in Figure 7.

**Experiment 2**

**Figure 8:**
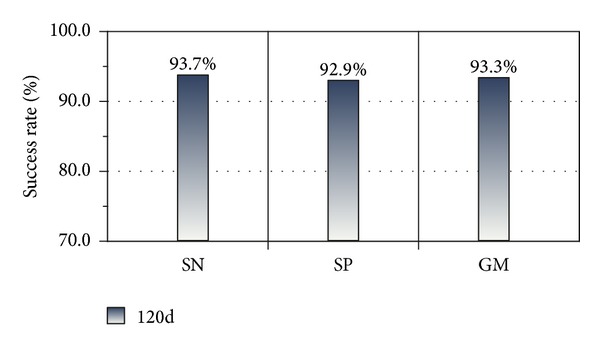
Testing results of original imbalanced testing set (120D features).

The imbalanced test set was tested by model_1_ achieved in Experiment 1, and the SN, SP, and GM values of the test results were calculated. The findings are shown in Figure 8.

The SN, SP and GM are 96.8%, 97.7%, and 97.2%, respectively, as shown in Figure 7. In addition, testing on the imbalanced testing dataset by model_1_ yielded favorable results, with SN, SP, and GM values of 93.7%, 92.9%, and 93.3%, respectively. These findings demonstrate that the classification works well.

To demonstrate that the performance of the 120D features we used is better than that of Cai's 188D features [17] for classifying cytokines, we conducted Experiments 3 and 4 and compared their effects. A comprehensive comparison of results illustrated the superiority of our method for cytokine identification.

**Experiment 3**

**Figure 9:**
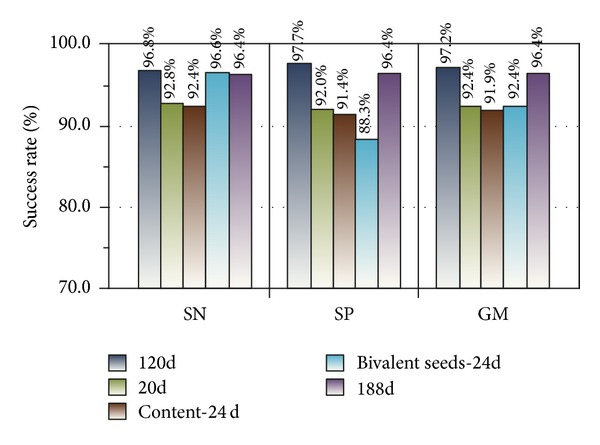
The comparison of 10-fold cross-validation results of five training sets.

We used five training sets with different properties by LibD3C. These sets included 120D, 20D, 24d (content), 24d (bivalent frequency), and 188D feature vectors. The method of obtaining 20D, 24d (content), and 24d (bivalent frequency) feature vectors is used to eliminate redundant attributes from the 188D feature vectors and preserve the required attributes utilizing Weka. The results were analyzed, and the five training models were saved as model_1_, model_2_, model_3_, model_4_, and model_5_. Model_1_ to model_5_ are shown in Table 1. Five groups of SN, SP, and GM values corresponding to the five training sets are shown in Figure 9. Five groups of feature vectors are detailed Section 2.

**Experiment 4**

**Figure 10:**
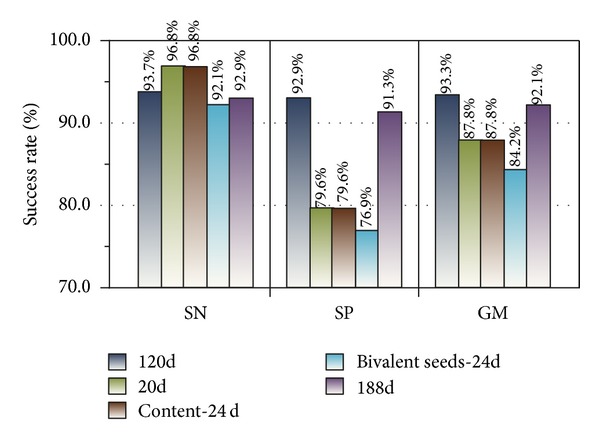
The comparison of testing results.

We tested the imbalanced testing dataset with model_1_, model_2_, model_3_, model_4_, and model_5_ in this order. The SN, SP, and GM values of the five testing results are shown in Figure 10.

The results show that the extraction method used in Experiments 3 and 4 is effective. The performance of the 120D feature vectors is better than that of the 188D feature vectors for the classification of cytokines. Thus, the 120D feature vectors are highly suitable for cytokine identification.

### 3.4. Performance of the Ensemble Classifier

To validate the classification effect of LibD3C, we conducted eight experiments (Experiments  5  to  12) using Weka (version 3.7.9). Experiment  5 includes the training and testing processes used in Experiments 1 and 2. The results of training and testing are shown in Figures 7 and 8, respectively.

We chose 7 simple classifiers from 18 basic classifiers from LibD3C for Experiments  6  to  12, which are similar to Experiment  5. These simple classifiers included RF, Libsvm, decision stump, SMO, naive Bayes, IB1, and J48, corresponding to Experiments  6,  7,  8,  9,  10,  11,  and  12, respectively.

The training model used for Experiment  5 was model_1_. Training models of Experiments  6  to  12  were model_6_ to model_12_, respectively, as shown in Table 2.

**Figure 11:**
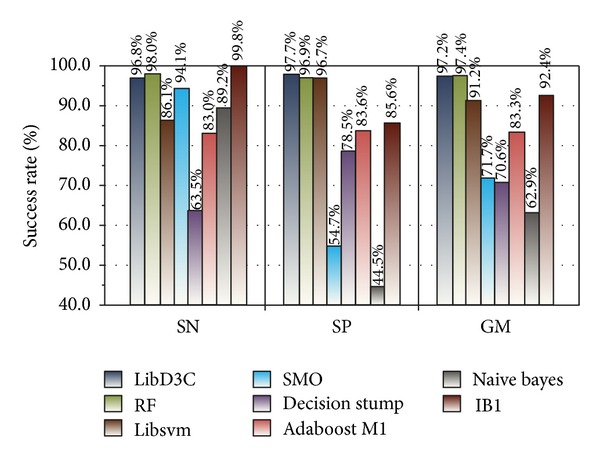
Performance comparison of 8 classifiers training on training set.

The 10-fold cross-validation results of ensemble classifier LibD3C and simple classifiers are shown in Figure 11. 

**Figure 12:**
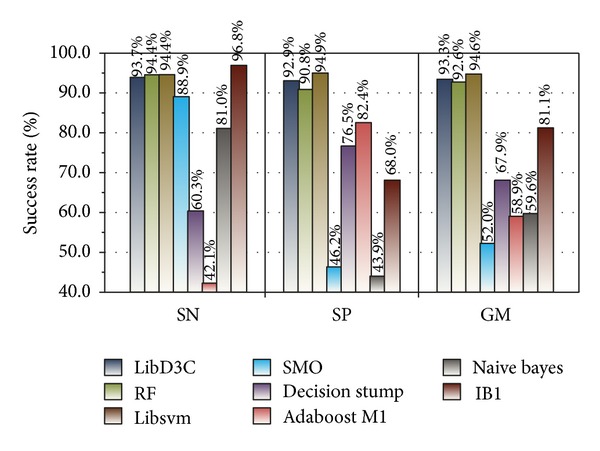
Performance comparison of 8 models testing on imbalanced testing set.

SN, SP, and GM values of the testing results are shown in Figure 12.

Figures 11 and 12 show the optimal performance of LibD3C based on dynamic selection clustering and circulating combination. The training results of LibD3C were 96.8%, 97.7%, and 97.2%, respectively, and SN, SP, and GM values of testing results reached 93.7%, 92.9%, and 93.3%, respectively. Compared with other simple classifiers, LibD3C has very high and stable SN, SP, and GM values.

### 3.5. Comparison with Other Softwares

There are just few software tools or web server available on line, which can predict cytokines from protein primary sequences. We develop a web server named CytoPre (Cytokine Prediction System) and compare it with CTKPred [11] and CytoKey (http://www.biomed.ecnu.edu.cn/CN/GPCR/Tools/BioAnalysistools/CytoRAP/CytoKey.aspx).

CTKPred was proposed for identifying cytokine using SVM. It extracted features from dipeptide composition and compared with Pfam searching. It was proved that CTKPred can outperform homologous searching, including HMM alignment. The sensitivity, specificity, and accuracy can get 92.5%, 97.2%, and 95.3%. CytoKey added amino acid composition and length features and gets 93.4%, 97.5%, 95.9% as sensitivity, specificity, and accuracy each.

**Figure 13:**
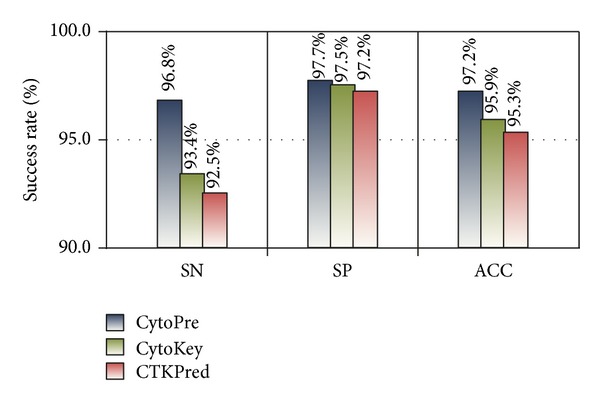
Performance comparison of 3 cytokine prediction systems.

We compared our CytoPre with CytoKey and CTKPred. Experiments showed that our system can outperforms the other two software, as shown in Figure 13, which suggested that the 188D protein composition and physical chemical properties features are more suitable for cytokine identification. Furthermore, the ensemble classifier can work better than single SVM.

### 3.6. Undiscovered Cytokines

We downloaded a total of 539616 protein sequences from the UniProt [19–21] database. Our goal was to predict all cytokines from whole protein sequences utilizing our training model. We detected 4151 candidate cytokine sequences (about 0.77%) from 539616 proteins. Of the 4151 candidate sequences, 39 were annotated as cytokines in UniProt. The other ones were done BLASTP to the known 16245 cytokines. Out of 4151 sequences, 444 showed regions with over 90% similarity to known cytokines, and another 697 sequences showed regions with over 50% similarity. The BLAST results and related data are supplied in the Supplementary Material (see Supplementary Material available online at http://dx.doi.org/10.1155/2013/686090) included in this paper.

Several conclusions may be made from the above experiments. First, not all of the cytokines have similar primary sequences. As well, BLAST is incapable of detecting all of the cytokines. Machine learning methods are necessary for detection. Finally, the experiments suggest that many cytokines have yet to be discovered. 

### 3.7. Discussion about the Experiments

Our preparatory work aimed to identify positive and negative families from the PFAM database. We then extracted the longest protein sequence in each family. To establish an effective classification model without deviation, we removed redundant sequences based on a sequence consistency standard. We extracted 120D feature vectors of positive and negative sequences based on the distribution of AAs with certain physicochemical properties and further sampled these to set up a training set. We then developed an ensemble classifier LibD3C to improve the stability and accuracy of cytokine classification. Cytokine identification was improved significantly in this paper in terms of accuracy and precision.

A series of experiments demonstrated the effectiveness of our method. We designed two group experiments to compare our methods (120D features) with Cai's (188D various features). The training results of our methods by LibD3C yielded SN = 96.8%, SP = 97.7%, and GM = 97.2%. In addition, the testing results of our methods were SN = 93.7%, SP = 92.9%, and GM = 93.3%. Two experimental sets of data generated by Cai's are SN = 96.4%, SP = 96.4%, and GM = 96.4%; SN = 92.9%, SP = 92.9%, and GM = 92.1%. The experimental results demonstrate that our method is superior to Cai's method in terms of classification validity because the hybrid approach may increase the weight of some information content and it is not conducive to all kinds of feature information extraction.

To prove that sampling has a significant influence on classification accuracy, we trained two groups of datasets by LibD3C. The first group used the test dataset (126 positive instances and 10588 negative instances) without sampling, while the second group used the rebuilding test dataset with SMOTE oversampling and *K*-means clustering undersampling, and they are extracted by 120D feature extraction algorithm. Experimentally, sampling is necessary to obtain good results. The SMOTE and *K*-means clustering algorithms were applied to small class and big class datasets, respectively. It avoids introducing excessive noise to the sampling set by SMOTE and effectively solves the problem of sample sparsity in the training set. In our approach, we employed a new type of ensemble classifier called LibD3C [32], which is a library for dynamic selection and circulating combination based on clustering. The ensemble classifier contained 18 basic classifiers and integrated some of these classifiers dynamically according to different objects of classification. Our goal is to achieve a classification result with the highest stability and accuracy. We developed eight groups of experiments to test the performance of LibD3C and conducted 10-fold cross validation using the rebuilding training set with LibD3C and seven basic classifiers. The results showed that the performance of the RT and Libsvm classifiers approached that of the ensemble classifier LibD3C. However, considering the sensitivity and specificity of the classifiers overall, LibD3C has obvious advantages.

Finally, we tested all protein sequences (539616) obtained from the UniProt database with the model trained through the method described prev and obtained 4151 cytokines. These cytokines are shown in the Supplementary Materials in FASTA format.

## 4. Conclusions

As a new interdisciplinary technology in the bioinformatics field, cytokine identification plays a very important role in the study of human disease. Studies that aim to improve the accuracy of cytokine prediction are of particular importance. To systematically present our experimental results and improve ease of use, we developed an online web server for cytokine prediction. Users input protein sequences that need to be predicted, and the server indicates which sequences are cytokines and displays geometric mean (GM) values of prediction. The results response to the HTML interface display whether it is cytokine and the prediction probability. The cytokine online prediction system can be accessed through http://datamining.xmu.edu.cn/software/CytoPre. The web site also provides related datasets and software for download.

## Supplementary Material

The supplementary material contains three files. The first file (4151.fasta) is the candidate sequences which were predicted by our method from UniProt. It contains 4151 sequences. The second file (16245.fasta) is all the cytokine sequences which is collected by us. The third file (blast.xlsx) is the blast result. 4151.fasta is chosen as the query file, and 16245.fasta is chosen as the database file. The blast.xlsx showed the similarity between the candidates predicted by our method and the known cytokines.

## Figures and Tables

**Algorithm 1:**
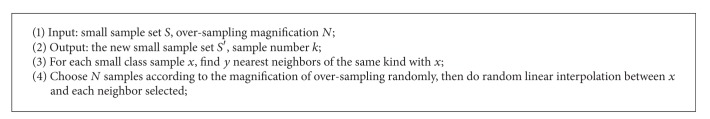
SMOTE over-sampling.

**Algorithm 2:**
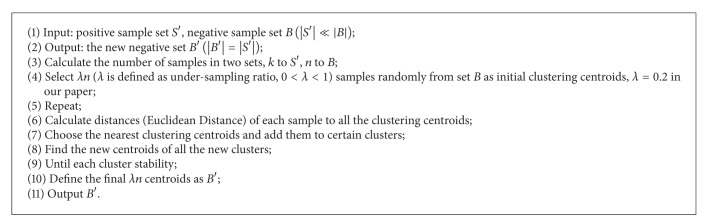
Under-sampling applies *K*-means clustering.

**Algorithm 3:**
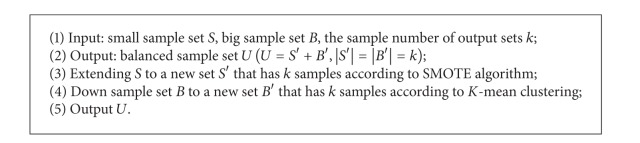
Ensemble algorithm of under-sampling combined with over-sampling.

**Table 1:** Training models of five training sets.

Features	Name of model	Save model
120d	Model_1_	LibD3C.model
20d	Model_2_	RF.model
24d (content)	Model_3_	Libsvm.model
24d (bivalent frequency)	Model_4_	SMO.model
188d	Model_5_	J48.model

**Table 2:** Training models of 8 classifiers.

classifier	Name of model	Save model
LibD3C	Model_1_	LibD3C.model
RF	Model_6_	RF.model
Libsvm	Model_7_	Libsvm.model
SMO	Model_8_	SMO.model
Decision stump	Model_9_	Decision stump.model
Naive Bayes	Model_10_	Naive Bayes.model
IB1	Model_11_	IB1.model
J48	Model_12_	J48.model
